# Why the elderly appear to be more severely affected by COVID‐19: The potential role of immunosenescence and CMV

**DOI:** 10.1002/rmv.2144

**Published:** 2020-07-15

**Authors:** Seilesh Kadambari, Paul Klenerman, Andrew J. Pollard

**Affiliations:** ^1^ Oxford Vaccine Group, Department of Paediatrics University of Oxford and the NIHR Oxford Biomedical Research Centre Oxford UK; ^2^ Peter Medawar Building for Pathogen Research University of Oxford Oxford UK

**Keywords:** CMV, COVID‐19, immunosenescence

## Abstract

The significantly higher mortality rates seen in the elderly compared with young children during the coronavirus disease 2019 (Covid‐19) pandemic is likely to be driven in part by an impaired immune response in older individuals. Cytomegalovirus (CMV) seroprevalence approaches 80% in the elderly. CMV has been shown to accelerate immune ageing by affecting peripheral blood T cell phenotypes and increasing inflammatory mediated cytokines such as IL‐6. The elderly with pre‐existing but clinically silent CMV infection may therefore be particularly susceptible to severe Covid‐19 disease and succumb to a cytokine storm which may have been promoted by CMV. Here, we evaluate the potential role of CMV in those with severe Covid‐19 disease and consider how this relationship can be investigated in current research studies.

**Abbreviations**
CMV: cytomegalovirus
Covid 19: coronavirus disease 2019
ICU: intensive care unit
IL‐6: Interleukin‐6
NHS: National Health Service
SARS‐CoV‐2: severe acute respiratory syndrome coronavirus 2

## 1 INTRODUCTION

The outbreak of coronavirus disease 2019 (Covid‐19) caused by the severe acute respiratory syndrome coronavirus 2 (SARS‐CoV‐2) appears to cause the highest morbidity and mortality in adults aged over 70 years.^1^, ^2^ One of the earliest and largest epidemiological studies in children with Covid‐19 showed that only 112 (5.6%) of 2143 children had severe disease (defined as hypoxia), 13 (0.6%) developed respiratory or multiorgan failure or acute respiratory distress syndrome (ARDS).^3^ In China, there have been no reports of children succumbing to Covid‐19. The reasons why children appear to develop less severe disease than adults is perplexing and likely multifactorial.

A range of environmental factors (greater transmission in cases who travel or in the workplace, increased contact with sick cases, cigarette smokers) and underlying health conditions (hypertension, diabetes and chronic respiratory disease) have been associated with severe Covid‐19 disease and death.^2^ All of these factors are more common in older adults than in children. The host immune response is also likely to play a pivotal role in accelerating disease progression in elderly individuals infected with SARS‐CoV‐2.

T cells have a crucial role in controlling viral infections. A central process in immunological ageing (also called immunosenescence) is reduction in thymic activity.^4^, ^5^ Cytomegalovirus (CMV) seroprevalence increases with age and approaches 80% by the age of 70 years in northern Europe.^6^, ^7^ In the elderly, CMV causes clonal T cell proliferation, reduction in naïve T cell diversity which in turn may lead to reduced capacity for immune responses to novel viral infections such as SARS‐CoV‐2. This article will consider the potential role of CMV in those with severe COVID‐19 disease.

## 2 CMV AND PREDISPOSITION TO RESPIRATORY VIRAL INFECTIONS

As aged T cells die, the thymus replenishes the T cell pool with naïve T cells. However, thymic output is reduced by 99% in 70 year olds compared with newborns.^8^ CMV infection drives compensatory memory T cell proliferation which ensures that overall T cell numbers do not decline significantly as we age. Very large CMV specific T cell responses in older people have parallels to the phenomenon of CMV induced T cell memory inflation seen in preclinical models.^9^, ^10^

The accumulation of terminally differentiated memory T cells and reduction in naïve CD8+ T‐cells in the elderly due to thymic involution is associated with influenza vaccine failure in older people.^11^, ^12^ A prospective Canadian study of residents aged over 65 years in 32 nursing homes found high T‐reg and high CMV reactive CD4+ T cells were predictive of risk of respiratory viral infections.^13^ In the OCTO and NONA Immune studies in Scandinavia, CMV seropositivity and the associated CD8 T cell expansions (inverted CD4:CD8 ratio) were linked to excess mortality in later life.^14^, ^15^ Although these findings are not universally consistent, they have been reproduced in other careful large scale epidemiology studies, for example, the EPIC study which associated high levels of CMV‐specific IgG with mortality.^16^ Although the impact of CMV on the CD8+ T cell compartment can be dramatic, whether CD8+ T cell expansion plays a direct causal role or is a marker for lack of immune control of CMV in such populations is not fully established.^17^

Multiple small cohort studies have attempted to evaluate the impact of CMV on the immunogenicity of influenza vaccines.^18^, ^19^, ^20^, ^21^ A study of 54 recipients of an intradermal influenza vaccine showed that CMV seropositivity was linked to reduced immune responses in individuals over the age of 60 years.^20^ The reduced effectiveness seen in this study appeared to be mediated by late differentiated CD4 T cells. A separate study in adults of all ages showed that CMV seropositive individuals over the age of 60 years had impaired B cell predictive biomarkers to influenza vaccine response.^18^ These data highlight that chronic CMV infections may have impaired humoral antibody responses. In contrast, a study of 731 residents in long term residential facilities showed that CMV serostatus did not influence pre or post influenza vaccination geometric mean antibody titer (GMT).^21^ Similarly, Wald and colleagues, showed that CMV serostatus had no impact on H1N1 influenza vaccine antibody responses in elderly individuals.^19^ Finally, a study of 348 individuals aged between 50 and 70 years, showed that CMV infection did not impair pneumococcal vaccine responses.^22^

**FIGURE 1:**
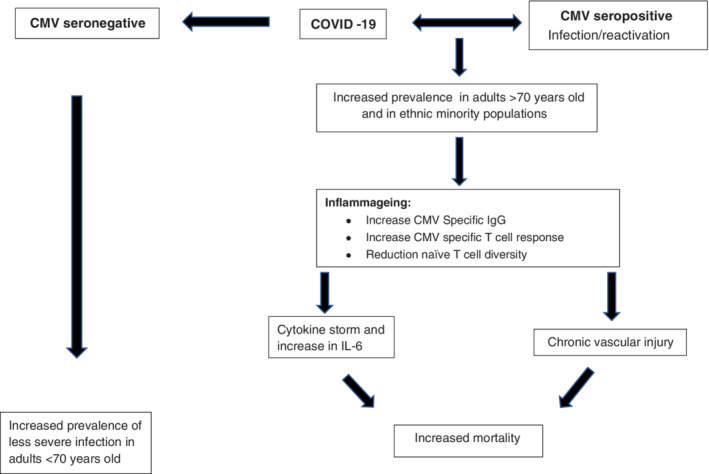
The association between Covid‐19, CMV and inflammageing which potentially leads to higher rates of Covid‐19‐related mortality in the elderly and in ethnic minority populations. Inflammageing is a condition characterised by elevated levels of blood inflammatory markers that carries high susceptibility to chronic morbidity, frailty and early death

Thus, overall, CMV seropositivity (with high levels of IgG) and the linked changes in bulk T cell populations appear to correlate with disease risk amongst the elderly (Figure 1). Assessment of CMV serologic and cellular immune responses in SARS‐CoV‐2 infected individuals and in healthy aged matched controls is required to assess whether there is a relationship between CMV infection (including reactivation) and severity of Covid‐19 disease. These data may also help us understand the potential effectiveness of any vaccine against SARS‐CoV‐2 in populations with high CMV seropositivity and thus inform future vaccine implementation.

## 3 CMV, COVID‐19 AND THE CYTOKINE STORM

Increased concentrations of IL‐6 have been seen in CMV infected individuals with poor responses to influenza vaccine.^12^ A longitudinal study of CMV DNA in peripheral monocytes in women over the age of 70 showed that CMV viraemic women had significantly higher IL‐6 levels (3.06 + 0.58 vs 1.19 ± 0.37 pg/mL, respectively, *P* < .001) than those without viraemia.^23^ Multiple studies have shown significant associations between IL‐6 and mortality in older adults.^24^, ^25^, ^26^ Schmaltz and colleagues showed a significant association between CMV seropositivity, IL‐6 and significant morbidity and mortality in elderly women over the age of 70 years old.^27^ The SALSA (Sacramento Area Latino Study on Aging) study, of Hispanic people aged over 60 years old in California, showed that high CMV IgG antibody levels were significantly related to mortality and that this was mediated by IL‐6.^28^

In 21 individuals with severe Covid‐19 disease there were increased cytokine levels (IL‐6, IL‐10 and TNFα), lymphopenia (in CD4+ and CD8+ T cells), and decreased IFNγ expression in CD4+ T cells are associated with severe Covid‐19.^29^ These findings have been supported in a larger study of 452 patients which showed reduction in the number of T cells in severe cases.^30^ Predictors of mortality from a retrospective, multicentre study of 150 confirmed Covid‐19 cases in Wuhan, China, included elevated ferritin (mean 1297.6 ng/mL in non‐survivors vs 614·0 ng/mL in survivors; *P* < .001) and IL‐6 (*P* < .0001), suggesting that mortality might be due to virally driven hyperinflammation.^31^ A randomised controlled trial showed that dexamethasone reduced mortality rates by a third in those individuals with severe Covid‐19 disease who required mechanical ventilation (29.0% vs 40.7%, RR 0.65 [95% CI 0.51‐0.82] (https://www.medrxiv.org/content/10.1101/2020.06.22.20137273v1). Tocilizumab, a recombinant human IL‐6 monoclonal antibody, has been used in Covid‐19 infected patients on the intensive care unit with anecdotal reports of some efficacy.^32^ The safety and efficacy of tocilizumab in the treatment of individuals with severe Covid‐19 disease is now the subject of a series of clinical studies (https://www.clinicaltrials.gov/ct2/show/NCT04322773, https://www.clinicaltrials.gov/ct2/show/NCT04315480?cond=tocilizumab&draw=2&rank=3, https://www.clinicaltrials.gov/ct2/show/NCT04306705?cond=tocilizumab&draw=2&rank=4 and https://www.clinicaltrials.gov/ct2/show/NCT04332913?cond=tocilizumab&draw=2&rank=5).

It is unknown whether severely affected individuals with Covid‐19, who develop a cytokine storm, are also infected with CMV or have CMV reactivation. CMV infection/reactivation may trigger the cytokine storm, play a contributory role or be an innocent bystander in individuals with severe Covid‐19 disease (Figure 1). If CMV does play a role in severity of Covid‐19 disease, individuals with latent infection, or reactivation, may benefit most from therapies directed against the inflammatory response. Analysis of CMV serostatus (with or without the associated immunologic profiling) is a straightforward assay to perform.

## 4 CMV, COVID‐19 AND ETHNICITY

Large observational cohort studies in the UK, USA and Holland over the last two decades show that CMV seroprevalence is independently associated with ethnicity.^33^, ^34^, ^35^, ^36^ A study conducted in London over 20 years ago showed 46% of white British women were CMV seropositive compared with 88% of Asian women and 77% of Afro‐Caribbean women.^33^ The most recent study in the UK showed that ethnic group and country of birth had greater influence on serostatus than household size or parity.^34^ This study showed CMV seroprevalence rates of 49% in white British women, 89% among south Asians born in the UK and 98% in women born in south Asia. In the USA, CMV seroprevalence rates amongst non‐Hispanic white people were 51% compared with 76% and 82% amongst non‐Hispanic Black and Mexican American groups.^36^

Preliminary surveillance data from the USA also highlight a similar trend in higher rates of Covid‐19 disease amongst people from ethnic minority backgrounds. During March 2020, in 99 counties across 14 states, ethnicity data were available for 580 out of 1482 laboratory confirmed cases.^37^ Overall, 45% (261) were non‐Hispanic white (white), 33% (192) were non‐Hispanic black (black), 8% (47) were Hispanic, 5%^32^ were Asian. Unpublished data from the UK show that 34% of 3883 patients admitted to intensive care units were from black or Asian ethnic minorities (https://www.icnarc.org/Our-Audit/Audits/Cmp/Reports). The OpenSAFELY study of factors associated with Covid‐19‐related hospital deaths, using linked electronic health records of 17 million adult National Health Service patients, showed a higher risk of death for black and Asian people which was only partially explained by pre‐existing clinical conditions or deprivation (https://doi.org/10.1101/2020.05.06.20092999).

CMV reactivation has been associated with all‐cause mortality and prolonged ICU stay in critically ill patients.^38^, ^39^ A placebo‐controlled randomised trial showed that ganciclovir/valganciclovir could significantly reduce CMV reactivation in such patients and potentially shorten duration of ICU stay.^40^ It is likely that the majority of Covid‐19 infected elderly ethnic minority patients are CMV seropositive at the time of developing disease. In patients with severe COVID‐19 disease understanding the role of CMV reactivation on the immune response may help identify novel treatment strategies to limit the potential inflammatory role of CMV.

## 5 CONCLUSION

Covid‐19 has disproportionately affected the elderly with significantly higher rates of mortality than in children. This observation has been recorded in every country affected by the pandemic. The rate of CMV seroprevalence increases with age. CMV has been shown to affect peripheral T cell phenotypes, increase inflammatory mediated cytokines such as IL‐6 and play a role in immune dysregulation as humans age. The role of CMV in those with severe Covid‐19 disease merits exploration through collection of CMV IgG, specific immune cells and viral load in observational cohorts of infected individuals. This may help inform our understanding of the use of immunomodulatory treatments such as monoclonal antibodies and convalescent sera, determine if CMV specific antiviral treatment has a role in the treatment of Covid‐19 individuals and potentially has implications for vaccine implementation.

## CONFLICT OF INTEREST

The authors have no competing interest.
